# Metagenomic Analysis of Respiratory RNA Virome of Children with and without Severe Acute Respiratory Infection from the Free State, South Africa during COVID-19 Pandemic Reveals Higher Diversity and Abundance in Summer Compared with Winter Period

**DOI:** 10.3390/v14112516

**Published:** 2022-11-14

**Authors:** Ayodeji E. Ogunbayo, Milton T. Mogotsi, Hlengiwe Sondlane, Kelebogile R. Nkwadipo, Saheed Sabiu, Martin M. Nyaga

**Affiliations:** 1 Next Generation Sequencing Unit and Division of Virology, Faculty of Health Sciences, University of the Free State, Bloemfontein 9300, South Africa; 2 Department of Biotechnology and Food Science, Durban University of Technology, P.O. Box 1334, Durban 4000, South Africa

**Keywords:** severe acute respiratory infection, virome, metagenomics, children, winter, summer

## Abstract

Viral respiratory infections contribute to significant morbidity and mortality in children. Currently, there are limited reports on the composition and abundance of the normal commensal respiratory virome in comparison to those in severe acute respiratory infections (SARIs) state. This study characterised the respiratory RNA virome in children ≤ 5 years with (n = 149) and without (n = 139) SARI during the summer and winter of 2020/2021 seasons in South Africa. Nasopharyngeal swabs were, collected, pooled, enriched for viral RNA detection, sequenced using Illumina MiSeq, and analysed using the Genome Detective bioinformatic tool. Overall, *Picornaviridae*, *Paramoxyviridae*, *Pneumoviridae*, *Picobirnaviridae*, *Totiviridae*, and *Retroviridae* families were the most abundant viral population in both groups across both seasons. Human rhinovirus and endogenous retrovirus K113 were detected in most pools, with exclusive detection of *Pneumoviridae* in SARI pools. Generally, higher viral diversity/abundance was seen in children with SARI and in the summer pools. Several plant/animal viruses, eukaryotic viruses with unclear pathogenicity including a distinct rhinovirus A type, were detected. This study provides remarkable data on the respiratory RNA virome in children with and without SARI with a degree of heterogeneity of known viruses colonizing their respiratory tract. The implication of the detected viruses in the dynamics/progression of SARI requires further investigations.

## 1. Introduction

Severe acute respiratory infection (SARI) is a leading cause of paediatric hospitalisation and mortality [1,2]. Respiratory RNA viruses are mostly implicated, with potential to cause pandemics [3]. Despite intensive laboratory investigations, a substantial proportion of acute respiratory infections are of unknown aetiology [4,5]. Beside the known viral agents often implicated in acute, symptomatic respiratory infections, recent analysis of the human respiratory virome has found hitherto undescribed viruses, viruses with unclear pathogenicity, viruses that induces symptoms but are relatively uncommon respiratory tract pathogens bacteriophages, and retroviral elements [6,7,8,9,10,11,12,13]. The virome composition is also reported to vary in response to environment, temperature/humidity, age, and immune status; and may be distinct in health and disease [12,14].

Recent studies suggests that the respiratory microbiome is linked to airway health and may influence the development of upper and lower respiratory tract illnesses (RTIs) [15,16,17]. Characterising detectable viral populations in the human respiratory tract is essential for comprehending the role of the respiratory virome to diseases affecting the respiratory tract, as this may reveal information on a large number of undetectable virus-induced RTIs [17,18]. Additionally, it may lead to the discovery of new viruses, the identification of various viral variations, and discovery of other viruses with hitherto undiscovered tropisms [17,18,19]. Moreover, analysis of the human respiratory virome may reveal differences in viral species between patients with high/low disease severity and healthy individuals [12,14,20]. Additionally, comprehensive data of viruses present in the respiratory tract of children during different weather seasons may provide baseline information on which pathogenic viruses are predominant in various weather conditions [19]. The knowledge garnered can guide seasonal surveillance, optimal schedule for prophylactics through vaccination, and the rational inventory and use of antivirals [21,22,23]. All of which can assist in prevention and control of RTIs.

The upscaled use of metagenomics next-generation sequencing (mNGS) has increasingly provided the invaluable comprehensive genomic profile of diverse micro-organisms present in clinical samples [14,24,25]. Beside detailed virome characterization, mNGS immediately offers additional information on virulence markers, epidemiology, and molecular genotyping [25]. Although the results from investigations of children’s respiratory virome in health and/or disease states have been reported [9,14,20,26,27,28], there is little information on the RNA respiratory virome and its composition during different weather seasonality in children with and without SARI. Therefore, using clinically informative data, this study provides pioneering comprehensive data on the respiratory virome of children in health and disease during varied weather conditions in the Free State Province of South Africa, with a view to afford baseline data that can guide further studies seeking to expand/understand viral respiratory diseases in children.

## 2. Materials and Methods

### 2.1. Study Settings and Demography

The patients were recruited from Botshabelo District Hospital, Pelonomi Regional Hospital, and National District Hospital in the Free State Province, South Africa. The patient population are children ≤ 5 years of age admitted with SARI (as per the World Health Organization (WHO) definition of SARI) and without SARI. The WHO case definitions for SARI were implemented as follows: children presenting with acute respiratory infection with a history of fever or measured fever of ≥38 °C and cough; with onset within the last ten days and requiring hospitalisation [29]. For this study, non-SARI children were defined as any child ≤ 5 years admitted/attended at the hospital for other forms of illnesses without any reported/documented history of illness with signs/symptoms relating to the respiratory tract in the last 14 days and at the time of admission/sample collection.

### 2.2. Sampling and Sample Collection

The sampling for this study was performed during two seasons, summer 2020/2021 and the winter of 2021. The summer sampling started from January to March 2020 and then commenced again between December 2020 to March 2021. This was due to national lockdown measures implemented during the COVID-19 pandemic. The winter sampling was performed between May 2021 to August 2021. In total, (n = 105 with SARI versus n = 80 without SARI) and (n = 44 with SARI versus n = 59 without SARI) were enrolled during the summer and winter periods, respectively. Upon admission, qualified medical personnel collected nasopharyngeal swabs (BD Diagnostics, Franklin Lakes, NJ, USA). After collection, the swab was immediately inserted into a viral transport media (VTM) (BD Diagnostics, Franklin Lakes, NJ, USA). Samples were labelled and transported to the University of the Free State-Next Generation Sequencing Unit (UFS-NGS Unit) Bloemfontein, Free State, South Africa, via cold chain transportation. Subsequently, the samples were registered, and stored at −80 °C until processing.

### 2.3. Sample Pooling

The respiratory samples collected per child (nasopharyngeal/oropharyngeal swabs) were processed as per in-house protocol. Briefly, the samples in VTM BD Diagnostics (Franklin Lakes, NJ, USA) were vortexed for 12 s and processed in pools using 1000 µL of each sample. For the summer samples, n = 105 children with SARI were processed into five pools (5 pools of n = 3, n = 8, n = 23, n = 55, and n = 16) and n = 80 children without SARI were processed into four pools (4 pools of n = 6, n = 16, n = 38, and n = 20 samples per pool). In winter, n = 44 children with SARI were processed into three pools (3 pools of n = 15, n = 14, and n = 15 samples per pool), and n = 59 children without SARI were also processed into three pools (3 pools of n = 20, n = 20, and n = 19 samples per pool). Beside the weather-guided sampling, pooling was further made based on the different waves of COVID-19 for the summer samples and based on monthly collection for the winter samples (considering the third wave was experienced through the winter periods). The adopted pooling strategy is presented in Supplementary Table S1.

### 2.4. Sample Processing, Enrichment and Extraction

During the summer season, the SARI group was processed into five pools (A1 to E1), and the non-SARI group was processed into four pools (A2 to D2). During the winter period, the SARI group was processed into three pools (E1 to G1), and the non-SARI group was processed into three pools (E2 to G2).

For each pool, the VTM was centrifuged at 10,000× *g* for 10 min to remove cellular debris. The supernatant was filtered through a 0.22 µm filter to remove the remaining possible host cellular debris and bacteria. The resulting filtrate was treated with a nuclease mixture of 0.1U µL^−1^ Turbo DNAse (Life Technologies, Carlsbad, CA, USA), 0.1U µL^−1^ RNAse One (Promega, Fitchburg, WI, USA), and 1X DNAse buffer (Life Technologies, Carlsbad, CA, USA) and incubation at 37 °C for 90 min, to remove non-incorporating nucleic acid. Nucleic acid extraction was performed using the PureLink viral RNA/DNA mini kit (Thermofisher Scientific, Waltham, MA, USA), following the manufacturer’s instructions but without using the carrier RNA. Extracted viral RNA was eluted in 50 µL of RNase-free water.

### 2.5. Positive and Negative Controls for Evaluation of Viral Recovery and Workflow Contamination

Clinical respiratory samples positive for RNA viruses (SARS-CoV-2 and rhinovirus A) (the viral transport media (VTM) tested positive for the different viruses during routine diagnostic testing and were provided by the National Health Laboratory Service, Universitas Bloemfontein, Free State, South Africa) and no-template control (Nuclease-free water) were subjected to nucleic acid extraction and processed through the same mNGS workflow as the pooled samples. The positive controls were included to evaluate the viral recovery of the workflow, and the negative control was included to assess the presence of cross-contamination or kitome contamination from reagents.

### 2.6. Qubit Quantification of the Extracted RNA

Extracted RNA was quantified using the Qubit RNA High Sensitivity (HS) Assay Kit (Thermofisher Scientific, Waltham, MA, USA) with the Qubit^®^ 3.0 Fluorometer (Thermofisher Scientific, Waltham, MA, USA) as per the manufacturer’s instructions.

### 2.7. DNase Treatment on Extracted RNA and Purification

Extracted RNA was treated with TURBO DNA-free™ reagents (Thermofisher Scientific, Waltham, MA, USA) according to the manufacturer’s instructions. The DNase-treated samples were purified using the RNeasy Mini Kit (Qiagen, Hilden, Germany) as per the manufacturer’s instructions.

### 2.8. Depletion of Ribosomal RNA (rRNA)

For each pool, rRNA depletion was performed to decrease the human, mouse, and rat rRNA by using NEBNext rRNA depletion kit (New England Biolabs, Ipswich, MA, USA) according to the manufacturer’s instructions.

### 2.9. Reverse Transcription and Whole Transcriptome Amplification

For library preparation purposes, the starting material was first converted to DNA. Therefore, the enriched, isolated viral RNA was reverse transcribed to generate cDNA. For the cDNA synthesis and random amplification, a QIASeq FX Single Cell RNA Library Preparation Kit (Qiagen, Hilden, Germany) was used as per the manufacturer’s instructions. Amplified cDNA was subsequently quantified using the Qubit™ 1X dsDNA HS Assay Kits with the Qubit 3.0 Fluorometer (Thermofisher Scientific, Waltham, MA, USA) as per the manufacturer’s instructions and normalised.

### 2.10. Library Preparation and Next-Generation Sequencing

Libraries were prepared using the QIASeq FX Single Cell RNA Library Kit (Qiagen, Hilden, Germany) according to the manufacturer’s instructions. The DNA libraries were analysed for fragment size distribution using the Agilent 2100 Bioanalyzer (Agilent Technologies, Santa Clara, CA, USA). Metagenomic sequencing was performed on the Illumina MiSeq system with the reagent kit v3 (Illumina, San Diego, CA, USA) for 600 cycles to generate 2 × 250 bp paired-end reads.

### 2.11. Bioinformatic Analysis

The generated paired-end reads were analysed using Genome Detective (https://www.genomedetective.com/, (accessed on 22 February 2022)), an automated web system for virus identification from high-throughput sequencing data [30,31]. The characteristic of this tool is presented in Supplementary Table S2. Confirmatory analyses were further performed for detections with low viral reads using BLAST.

### 2.12. Phylogenetic Analysis

To analyse the genetic variation in selected respiratory viruses, nucleotide sequences with close to full genome coverage detected in this study (at least 99%) were compared with hits entries in the GenBank database. Two Human rhinovirus (HRV) representative sequences, HRV A C-series and HRV A B-series, with 99.2% and 99.8% genome coverage, respectively, were selected. For the HRV analysis, the 100 closest hits full-length genomes of HRV A were retrieved for analysis, and a full-length genome of HRV B was also retrieved for use as an outgroup. Phylogenetic analysis was conducted using the neighbor-joining method using MEGA 6.0 with a bootstrap value of 1000 [32].

### 2.13. Statistical Analysis

Continuous variables are reported as median, whereas categorical variables are reported as percentages and calculated using Microsoft excel version 365 version 2206.

## 3. Results

### 3.1. General Demographics and Clinical Presentations

The average age for the SARI and non-SARI groups were 14.5 and 19 months, respectively. A total of 27 (18.1%) and 12 (8.5%) in the SARI and non-SARI group had HIV infection, respectively. Notably, none of the SARI group required the need for intensive care unit. Complete demographic and clinical data are presented as Supplementary Table S3.

### 3.2. Sequencing Data

After quality filtering, the total number of reads per pool during summer season ranged from 140,444 to 1,703,176. The proportion of viral reads in each pool ranged from 2% to 12% (Table 1). During winter season, the total number of reads per sample pool after quality filtering ranged from 13,308 to 293,394 and the proportion of virus-specific reads in each pool ranged from 2% to 9% (Table 1). It was noteworthy that the highest percentage of viral reads were observed in the non-SARI groups across the two investigated seasons (Table 1).

### 3.3. Respiratory Tract Virome Analyses from Children with and without SARI during Summer and Winter

**Figure 1:**
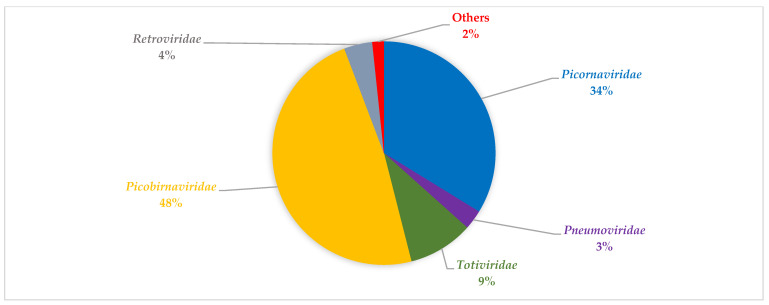
An overview of representative viral family distribution from children with SARI in summer based on read abundance. The viral families shown include, *Picobirnaviridae*, *Picornaviridae*, *Pneumoviridae*, *Totiviridae*, and *Retroviridae*. Others comprise of *Partitiviridae*, *Betaflexiviridae*, *Coronaviridae*, *Chrysoviridae*, *Virgaviridae*, *Herpesviridae*, *Phycodnaviridae*, *Endornaviridae*, and *Circoviridae*.

**Figure 2:**
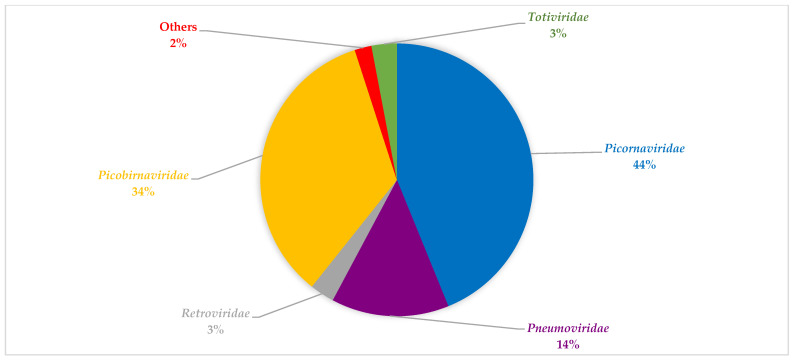
An overview of representative viral family distribution from children with SARI in winter based on read abundance. Viral families with the most abundance include *Picornaviridae*, *Pneumoviridae*, *and Picobirnaviridae*, *Retroviridae*, and *Totiviridae*. Others include *Endornaviridae*, *Coronaviridae*, *Virgaviridae*, and *Tombusviridae*.

Bioinformatic analyses revealed diverse viruses in the respiratory tracts of children with SARI across the two seasons investigated (Table 2 and Table 3, respectively). During summer season, viral families with the most abundant viral reads can be grouped into: *Picornaviridae*, *Retroviridae*, *Totiviridae*, *Picobirnaviridae,* and *Pneumoviridae* (Figure 1). During winter, the viral family with the most abundant reads can be grouped into *Picornaviridae*, *Pneumoviridae*, and *Picobirnaviridae* (Figure 2). Notably, the *Betaflexiviridae*, *Partitiviridae*, *Chrysoviridae*, *Herpesviridae*, *Phycodnaviridae*, and *Circoviridae* were exclusively detected in the summer period in this group.

**Figure 3:**
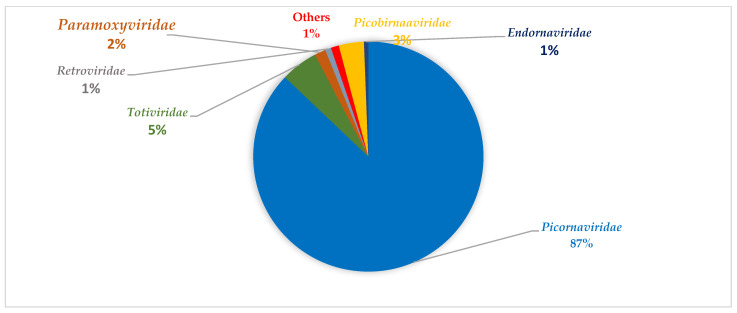
An overview of representative viral family distribution from the non-SARI group in summer based on read abundance. The major viral families detected include *Picornaviridae*, *Totiviridae*, *Paramoxyviridae*, *Retroviridae*, *Picobirnaviridae*, and *Endornaviridae*. Other viral families at low abundance include *Coronaviridae*, *Partitiviridae*, *Chrysoviridae*, and *Herpesviridae*.

**Figure 4:**
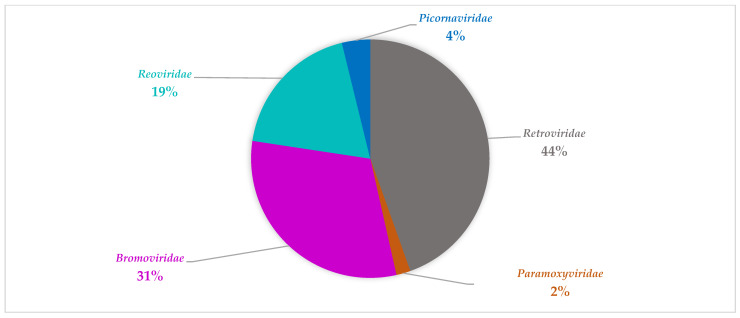
An overview of representative viral family distribution from the non-SARI group in winter based on read abundance. The viral families include *Retroviridae*, *Bromoviridae*, *Reoviridae*, and *Picornaviridae*, and *Retroviridae*.

The viral composition in the non-SARI group also contains diverse viral families (Table 4 and Table 5). The viral families with the most abundant reads in this group during summer can be grouped into *Picornaviridae*, *Totiviridae*, *Paramoxyviridae*, *and Retroviridae* (Figure 3). During winter, the viral families with the most reads in this group were mainly *Retroviridae*, *Bromoviridae*, and *Reoviridae* (Figure 4). Notably, the *Reoviridae* and *Bromoviridae* viral families were only detected in this group during winter period. Comparatively, *Totiviridae*, *Coronaviridae*, *Partitiviridae*, *Picobirnaviridae*, *Endornaviridae*, and *Herpesviridae* were exclusively detected during the summer period.

### 3.4. Viral Composition during the Seasons Investigated and between Both SARI and Non-SARI Group

**Figure 5:**
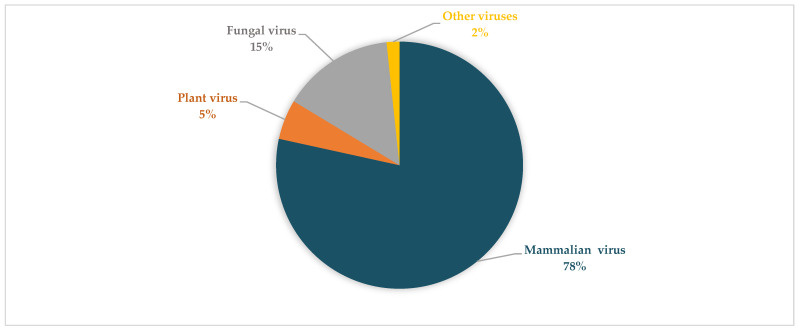
Different types of viruses from the SARI group in winter and summer. Mammalian virus were the most predominant followed by fungal, plant, and a small fraction of other viruses (avian viruses, yeast viruses, and algae viruses).

**Figure 6:**
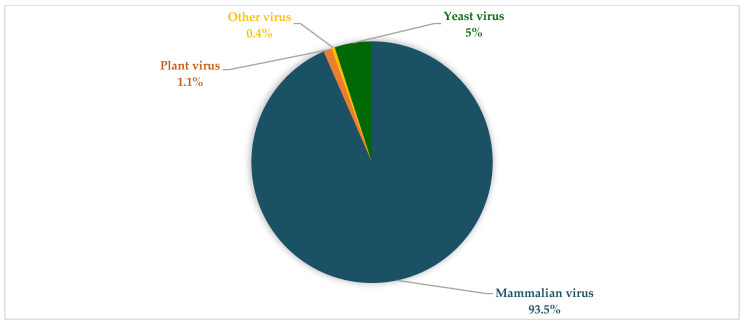
Different types of viruses from the non-SARI group in winter and summer. Mammalian viruses were predominant, followed by yeast virus, plant virus, and a small fraction of avian, fungal, and algae viruses.

Based on viral reads, mammalian eukaryotic viruses were more abundant than plant and other viruses in both groups during the two seasons (Figure 5 and Figure 6). More so, HRV and human endogenous retrovirus K113 were the only viruses detected in most pools from the SARI and non-SARI groups during both the summer and winter seasons. Comparatively, regardless of having SARI or not, children in the summer group (both SARI and Non-SARI) had higher viral reads and abundance than the participants recruited during the winter period. Notably, the *Pneumoviridae* viral family (RSV) was only detected in the SARI group during both periods investigated. Moreover, viral reads for Human Rotavirus and Human parainfluenza viruses 2 and 3 were exclusively detected in the non-SARI group. Besides detection of reads matching human and eukaryotic viruses, several phage-related reads (matching Escherichia phage, Enterobacteria phage, Streptococcus phage, Klebsiella phage, Staphylococcus phage, Stx2-converting phage, Lactococcus phage, Vibrio phage, Salmonella phage, Proteus phage, and Aggregatibacter phage) were detected from both groups (Supplementary Table S4).

### 3.5. Exclusive Viral Reads Detection in Pools

Beside the detection of RSV exclusively in patients with SARI. Its exclusive detection was also noted between second and third waves upward (Table 2). Rotavirus was also exclusively detected only in the July/August Non-SARI samples (Table 5). Of interest, the only SARS-CoV-2 reads detected were from the pools between COVID-19 lockdown declarations through the first wave.

### 3.6. Viral Recovery and Evaluation for Contaminants in the mNGS Workflow

As presented in supplementary data (Table S5 and Figure S1a,b), the recovery and distribution of reads on the viruses included in the run as positive control suggest that RNA viral genomes can be successfully obtained through the workflow adopted in this study. Notably, only bacteriophages were detected at less than 0.6% genome coverage in the no template control included in the sequencing run.

### 3.7. Phylogenetic Analysis

**Figure 7:**
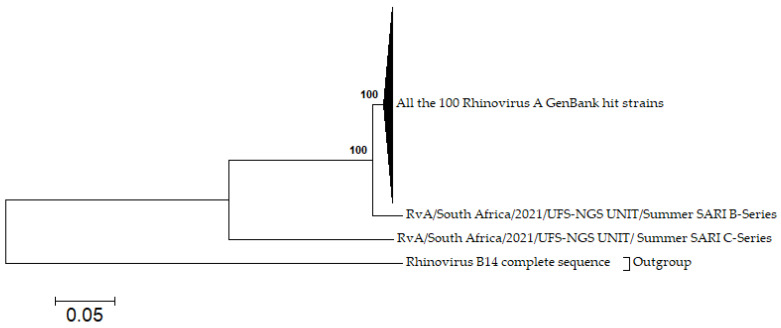
Phylogenetic analysis of rhinovirus A strains (B series and C series SARI) with 100 hit HRV-A strains from the GenBank. The complete 100 rhinovirus A hits strains were collapsed and are available in supplementary Figure S2.

The phylogenetic analysis for the contigs built identified a distinct type of human rhinovirus A named (RvA-Cseries) which formed a distinct cluster with the 100 closest hits on GenBank (mostly sequences isolated in 2021), while RvA-Bseries formed a moderate cluster with the closest hits on GenBank. The RvA-Bseries series exhibited nucleotide/amino acid similarities with the RvA-Cseries ranging of 70.6%; 64.7%. In comparison with the 100 hits from GenBank, the RvA-Bseries displayed nucleotide and amino acid identities of 95.7–96.0% and 98.0–98.3% respectively, while the RvA-Cseries exhibited nucleotide and amino acid identities of 70.6–72.7% and 65.9–66.1%, respectively. The 100 HRV A hits from GenBank are collapsed in Figure 7 but complete data are provided in Table S3. The two complete genomes of HRV A (RvA-Cseries SARI and RvA-Bseries SARI), and one partial genome of HRV C (RvC-Cseries SARI) sequences were logged in the GenBank (Accession numbers: OP114090; OP114091; OP114092).

## 4. Discussion

This study demonstrated how mNGS is an important tool to decipher the respiratory RNA virome with significant depth. The diversity of detected reads originating from ~59 species in the pooled samples in this study mirrors the relative ease of exposure of the human respiratory system to several organisms. The majority of known respiratory RNA viruses detected in this study belonged to four families, namely *Picornaviridae*, *Pneumoviridae*, *Coronaviridae*, and *Paramoxyviridae*, and interestingly, these families have all been previously detected in the human respiratory tract [14,18,27,28]. The detected reads for several plants and animal viruses and other human non-respiratory viruses in this study for which pathogenicity in the respiratory tract is yet to be established can be either regarded as temporary commensals, or could have been introduced from the environment, contact with animals, food, and water [26,28]. While this study further reports on viral reads for several RNA viral families/species which have not been reported elsewhere, some viruses reported from other studies were not detected. For instance, some studies [14,33] detected a high level of viruses in the family *Anelloviridae*, which were not detected in the current study. The differences noted in viral detection in different studies may be attributed to differences in geographical location, viral exposure, or methodological choices [18].

In terms of virome composition in children with and without SARI, regardless of the sampling period (winter or summer), the proportion and abundance of viruses (in terms of family diversity and reads number) in the SARI group were higher than that of the control group. This observation is consistent with previous studies [14,20] on the virome composition in febrile versus afebrile children. Specifically, a study by Wylie et al. [20] reported higher viral sequences in the nasopharyngeal swabs samples from febrile children compared with afebrile ones. Wang et al. [14] also reported higher viral abundance in the respiratory tracts of children with SARI compared with children without SARI. Similar to other metagenomic studies [14,28], and PCR-based studies [34,35], the samples from the subjects in this study without SARI also contains reads from known epidemic respiratory viruses, such as HRV A, B, and C, Coronavirus NL63, PIV 2, and 3. The presence of these viruses in children without SARI may be transient, or the particular viral strain does not induce clinical symptoms, especially with HRV, which has been reported to be commonly present in the respiratory tracts of young children [20,36,37,38,39,40]. Moreover, the detection of the known pathogenic viruses in the non-SARI group further makes it challenging to use the virome data in establishing/judging the possible causative agents (in the SARI group) in this study as previously performed [14]. Nonetheless, observations from this study highlight the need for comprehensive studies of the dynamics of the airway virome over time to evaluate the possibility of a transient infection becoming symptomatic and to understand the future contribution of asymptomatic viral infection to community transmission.

Regarding the contribution of HIV to virome composition, a previous study reported alteration of microbiome composition in individuals with advanced HIV infection [41], while Beck et al. [42] reported that the respiratory microbiome measured in whole bronchoalveolar lavage (BAL) was indistinguishable between an HIV-infected and uninfected population. Similarly, Monaco and colleagues reported that in the absence of immunodeficiency, HIV has a minimal effect on the enteric DNA virome and bacterial microbiome. Rather, AIDS and the resultant immunodeficiency were associated with notable alterations [43]. In the current study, even though samples were pooled regardless of HIV status, the HIV-infected children were on antiretroviral therapy (ART), and as such their inclusion is not expected to contribute to alteration in the pooled virome composition towards increased viral detection.

Furthermore, the detection of sequence reads belonging to the human endogenous retrovirus K113 (HERV-K) in this study can be attributed to the recent entry of this group into the human genome. The group includes numerous retroviruses with full-length intact proviruses [44]. Typically, these proviruses only occasionally express in patients with cancer or autoimmune disease [45,46]. However, a recent study by Ferravente and colleagues used NPS to characterise the respiratory virome in SARS-CoV-2 patients; they detected HERV-K in 10 patients who were characterised with severe outcomes [47]. Similarly, Temerozo et al. [13] characterised the virome of tracheal aspirates of severe COVID-19 patients and linked the detection of HERV-K in their lower airways with early mortality. In this study, the detection of reads for the HERV-K113 in the sample pools for both SARI and non-SARI cases during winter and summer cannot be exclusively associated with severe outcomes, especially in patients in the SARI group, and as such the detection of this provirus in both groups may warrant further studies.

The detection of HRV in most sample pools in both SARI and non-SARI cases throughout the sampling period (winter and summer) further corroborates reports from another study in South Africa [48] and other countries [49,50,51] that HRV is an all-year-round virus that may circulate in both winter and summer. The detection was reportedly maintained or heightened post-COVID-19-targeted NPIs [52,53]. In addition, we identified a distinct type of HRV A, which from the phylogenetic analysis formed a distinct cluster with the closest hits on GenBank. These closest hits were majorly samples detected and deposited in the year 2021 from the USA. Despite being the closest 100 hits on GenBank, the detected HRV A strain only had a nucleotide/amino acid identity similarity that ranged from 70.6–72.7% to 65.9–66.1% with these hits. This detection may warrant future genomic surveillance of HRV in the study setting. Although, the presence of RSV in healthy children has been reported previously [14,18,28], in this study, reads for RSV were exclusively detected only in the SARI pools. More so, the total absence of the seasonal influenza virus was noted and can be due to the sampling period (during COVID-19 waves), where the complete absence/limited presence of influenza viral circulation has been reported [54,55].

In terms of viral diversity/abundance during the summer versus winter period, both the SARI and non-SARI groups had a relatively higher viral diversity and abundance (in terms of reads number) of known respiratory viruses and other viruses during the summer than the winter period. The differences in viral abundance can be due to the number of children recruited during the winter period, as fewer children presented to the hospital with SARI and non-SARI cases during this time, resulting in fewer participants per pool, thus less viral abundance. It can also be due to viral exposure, considering the sampling period, where several non-pharmaceutical interventions (NPIs) such as wearing of mask and constant hand sanitization were in place. Moreover, there was a build-up of social restrictions alongside other NPIs into the winter period which may have affected viral exposure and circulation, especially of respiratory viruses. Hence, there was a low pocket of circulating and limited detection of respiratory viruses in the winter period.

Beside the weather-based sampling, the samples were also pooled based on pre and during waves of COVID-19 experienced in the country (for the summer samples) and based on months (for the winter samples). The rationale was to evaluate compositional changes, if any, in the respiratory virome prior to COVID-19 declaration, over the different COVID-19 waves and different winter months. Notably, no RSV reads were detected in the SARI sample pools prior to COVID-19 lockdown declaration through the first wave. Reads for RSV were only detected from second wave upwards. This agrees with a previous study [56] where the surge in reported cases of RSV were noted from summer 2020 (during second wave). Furthermore, the only reads detected for SARS-CoV-2 were from the pools between COVID-19 declaration through first wave. Reads for SARS-CoV-2 would be expected in at least more than one pool considering the study period; however, relatively fewer reads for SARS-CoV-2 were detected. From our recent reports on the pathogen profile of children with SARI using a multiplex real-time PCR in the same study settings and period, comparatively higher Ct values (average of ≥33) were generally noted for SARS-CoV-2 (inversely correlated with low viral load) in the child population [57]. If this is also the case across the child population in this study in those possibly infected with COVID-19, then suggestively SARS-CoV-2 viral reads recovery could have been impacted or even masked by the more abundant host reads and could have resulted in missed detection by mNGS [58,59,60], hence fewer reads for SARS-CoV-2. Moreover, Ct values of ≤30 were previously reported for optimal detection of SARS-CoV-2 virus in whole genome sequencing [61].

A major limitation of the study was the inability to attribute the occurrence of each virus to individual participants due to the pooling of samples. In this regard, a larger individual-based longitudinal sampling can be performed. This would allow a comprehensive elucidation of the length of stay of these viruses in the respiratory tract in health and disease, and the existence or not of a permanent viral community in the respiratory niche in each patient. Moreover, relatively fewer participants were recruited during the winter period, a situation beyond control as fewer admissions were seen for both SARI and Non-SARI cases. Furthermore, this study only focused on enrichment for RNA viruses and thus cannot account for missed DNA viruses; thereby necessitating the need for further studies on the DNA respiratory virome in children with and without SARI. Lastly, using a 0.22 µm filter may have resulted in the loss of larger viruses that could have been detected. Additionally, the non-SARI group included diverse groups of patients, with varying conditions including meningitis, neurological disorders, and diarrhoea and may thus present with detectable viruses (such as enterovirus and rotavirus) in their nasopharynx. In this regard, some virome characteristics described in the non-SARI group may be confounded by the diversity of some disorders in these patients.

## 5. Conclusions

This study provided remarkable pioneering data of the RNA respiratory virome of children with and without SARI in South Africa during winter and summer when the COVID-19 pandemic was at the peak before vaccination interventions. The diverse distribution of viral reads in each sample pool, highlights similarities and differences both within and between children with and without SARI. Despite the study’s exploratory nature, it raised concerns about whether some of the viruses detected with unknown pathogenicity may exacerbate clinical course or contribute to symptoms manifestation. It also raises the question of whether the several known epidemic viruses detected in the non-SARI group are transient or biomarkers for future respiratory infection. Moreover, the detection of a distinct HRV A type in this study highlights the need for continuous genomic surveillance of the respiratory virome for possible detection and characterization of other distinct/novel strains which may have the potential for outbreak.

Of importance, considering the study sampling period, the virome composition could have been impaired due to various NPIs against COVID-19 such as restriction of social gatherings and wearing of mask; all of which significantly impacted viral circulation of respiratory viruses. Consequently, there may be a need for other studies to evaluate the spectrum/composition of respiratory virome in health and disease post-COVID-19; of which this study would serve as an invaluable baseline. Lastly, the detection of expected/unexpected pathogenic viruses in both SARI and non-SARI groups, the detection of viruses with potential to worsen the course of respiratory infection, and the detection of other pathogenic viruses with unknown roles in respiratory infection further highlights the complexity of this niche in health and disease and may contribute further to the understanding and management of SARI in children.

## Figures and Tables

**Table 1:** Reads for each pooled samples after filtering with the percentage of viral reads.

Pooled Samples	Number of Samples	Reads after Quality Filtering	% of Viral Reads
SARI Summer			
SARI Pool A1	3	1,079,760	2
SARI Pool B1	8	1,703,176	3
SARI Pool C1	23	8,500,586	4
SARI Pool D1	55	1,341,384	5
SARI Pool E1	16	862,754	11
Non-SARI Summer			
Non-SARI Pool A2	6	140,444	5
Non-SARI Pool B2	16	312,496	12
Non-SARI Pool C2	38	366,146	4
Non-SARI Pool D2	20	1,250,024	6
SARI Winter			
SARI Pool E1	15	293,394	2
SARI Pool F1	14	166,726	2
SARI Pool G1	15	272,596	3
Non-SARI Winter			
Non-SARI Pool E2	20	15,434	5
Non-SARI Pool F2	20	13,308	6
Non-SARI Pool G2	19	37,010	9

SARI: severe acute respiratory infection.

**Table 2:** Viral family, species, reads numbers, and genome structure of viruses detected in the respiratory sample pools of children with SARI during summer period. **Number of reads/coverage %**

Family	Viral Species/Read Number	Genome Structure	A1	B1	C1	D1	E1
***Picornaviridae***	HRV-A **(21,328)**	ssRNA	342/16.2	15,441/99.8	5495/99.2	22/13.6	28/3.4
	HRV-C **(9972)**	ssRNA	1122/53.9	2315/25.2	31/8.2	264/25.8	6240/89.5
	Parechovirus **(236)**	ssRNA		176/27.8	60/8		
	Enterovirus J **(60)**	ssRNA			60/7.9		
	HRV-B **(261)**	ssRNA				159/28.3	102/4.1
	Enterovirus A **(44)**	ssRNA				44/8.4	
	Enterovirus B **(2523)**	ssRNA					2523/82.8
	Enterovirus C **(13)**	ssRNA					
	Enterovirus A114 **(101)**	ssRNA		24/3.4		77/30.1	13/3
***Partitiviridae***	Fig cryptic virus **(420)**	dsRNA		420/78.1			
***Pneumoviridae***	RSV-B **(2333)**	ssRNA			234/23	1840/89.9	493/48
	RSV-A **(572)**	ssRNA			124/19	522/40.5	50/7.6
***Totiviridae***	Scheffersomyces segobiensis virus L **(7892)**	dsRNA	152/49.4	271/45.1	1395/46.3	3110/84.4	2964/80.3
	Diatom colony-associated dsRNA virus 3 **(27)**	dsRNA			27/5.2		
	Diatom colony-associated dsRNA virus 10 **(21)**	dsRNA			17/7.3		4/4.5
	Red clover powdery mildew-associated totivirus 2 **(218)**	dsRNA				198/41.5	20/3.5
	Xanthophyllomyces dendrorhous virus L1B **(82)**	dsRNA				82/16.9	
	Saccharomyces cerevisiae virus **(52)**	dsRNA				52/19.2	
	Maize-associated totivirus 2 **(1346)**	dsRNA					1346/11.8
	Red clover powdery mildew-associated totivirus 6 **(3)**	dsRNA					3/3.5
***Picobirnaviridae***	Otarine Picobirnavirus (segment 2) **(49048)**	dsRNA		18/52			49,030/98.4
	Picobirnavirus green monkey/KNA/2015 **(31)**	dsRNA		6/24.9		25/6.9	
	Picobirnavirus dog/KNA/2015 **(125)**	dsRNA				125/29	
	Human Picobirnavirus **(75)**	dsRNA				75/12.5	
***Coronaviridae***	SARS-CoV-2 **(2)**			2/1			
***Retroviridae***	Human endogenous retrovirus K113 **(1287)**	ssRNA-RT	208/17.2	109/16.8	107/16.1	784/22.5	79/20
	Moloney murine leukaemia virus **(1528)**	ssRNA	120/13.7	1404/22.4		4/7	
	Equine infectious anaemia virus **(651)**	ssRNA		28/4.8	107/16.1	404/20	112/19
	Koala retrovirus **(2)**	ssRNA-RT	2/4.3				
	Reticuloendotheliosis virus **(489)**	ssRNA-RT	61/9.6			428/13.2	
	RD114 Retrovirus **(13)**	ssRNA-RT				13/5.8	
	Chick syncytial virus **(3)**	ssRNA-RT			3/23.9		
	Atlantic salmon swim bladder sarcoma virus **(79)**	ssRNA-RT				79/8.4	
	Feline leukaemia virus **(82)**	ssRNA				70/7	12/4.7
	Baboon endogenous virus strain M7 ssRNA **(14)**	ssRNA-RT				14/4.5	
	Friend murine leukaemia virus **(31)**	ssRNA					31/5.1
	Gibbon ape leukaemia virus **(14)**	ssRNA					14/3.7
	Bovine retrovirus CH15 **(9)**	ssRNA-RT					9/8.7
***Chrysoviridae***	Penicillium chrysogenum virus segment 3 **(2)**	dsRNA	2/2.7				
***Virgaviridae***	Tobacco Mosaic Virus **(60)**	ssRNA	60/10.2				
	Pepper mild mottle virus **(3)**	ssRNA	3/1.1				
***Betaflexiviridae***	Apple chlorotic leafspot virus **(622)**	ssRNA				527/41.4	95/18.4
	Apple stem grooving virus **(33)**	ssRNA				33/6.6	
***Herpesviridae***	Saimiriine gammaherpesvirus 2 **(2)**	dsDNA			2/0.3		
***Phycodnaviridae***	Micromonas pusilla virus 12T **(26)**	dsDNA				26/0.1	
***Endornaviridae***	Grapevine endophyte alphaendornavirus **(94)**	ssRNA				94/10.2	
	Bell pepper alphaendornavirus **(4)**	ssRNA				4/2.4	
***Circoviridae***	Porcine stool-associated circular virus **(16)**	ssDNA				16/19	
**Unclassified**	Bovine serum-associated circular virus **(331)**	Unknown	28/99.2				303/99.8

HRV-A: human rhinovirus A; HRV-C: human rhinovirus C; HRV-B: human rhinovirus B; RSV: respiratory syncytial virus; SARS-CoV-2: severe acute respiratory syndrome coronavirus 2.

**Table 3:** Viral family, species, reads numbers, and genome structure of viruses detected in the respiratory sample pools of children with SARI during winter period. **Number of reads/coverage %**

Family	Viral Species/Read Number	Genome Structure	E1	F1	G1
***Picornaviridae***	HRV-C (231)	ssRNA	130/30.9	69/26.5	32/7.5
	HRV-A (234)	ssRNA	6/3.5	26/13	206/52
	Enterovirus B (126)	ssRNA		31/11.4	95/30.7
***Pneumoviridae***	RSV-B (186)	ssRNA	126/18.7	15/5.5	145/16
	RSV-A (1)	ssRNA		1/0.7	
***Retroviridae***	Human endogenous retrovirus K113 (39)	ssRNA-RT	11/1.8		28/8.4
***Picobirnaviridae***	Otarine Picobirnavirus (segment 2) **(9)**	dsRNA	9/15.3		
	Picobirnavirus green monkey/KNA/2015 **(325)**	dsRNA			325/82
	Chicken Picobirnavirus (segment RNA 1) **(126)**	dsRNA			126/17.5
***Endornaviridae***	BPA **(6)**	ssRNA	5/0.6	1/0.4	
***Coronaviridae***	Human coronavirus NL63 **(3)**	ssRNA	3/0.8		
***Virgaviridae***	Tobacco mosaic virus **(6)**	ssRNA		6/5.4	
***Retroviridae***	Equine infectious anaemia virus **(4)**	ssRNA		2/3.7	2/1.8
***Totiviridae***	Scheffersomyces segobiensis virus L **(40)**	dsRNA			40/10.2
***Tombusviridae***	Bermuda grass latent virus **(8)**	ssRNA			8/4.5

HRV-C: human rhinovirus C; HRV-A: human rhinovirus A; RSV A: respiratory syncytial virus A; RSV-B: respiratory syncytial virus B; BPA’: bell pepper alphaendornavirus; SSVL: scheffersomyces segobiensis virus L.

**Table 4:** Viral family, species, reads numbers, and genome structure of viruses detected in the respiratory sample pools of the non-SARI group during summer period. **Number of reads/coverage %**

Family	Viral Species/Read Number	Genome Structure	A2	B2	C2	D2
***Picornaviridae***	HRV-C (**7926**)	ssRNA	1388/85.5	6495/93.3	31/8.2	12/1.2
	HRV-A (**29420**)	ssRNA	726/53.1	27923/92.9	746/86	25/4.2
	HRV-B (**347**)	ssRNA	347/65.8			
	Enterovirus J (**71**)	ssRNA			60/7.9	11/1.0
	Enterovirus B (**513**)	ssRNA				513/51.2
	Enterovirus A (**17**)	ssRNA				17/9
***Totiviridae***	Scheffersomyces segobiensis virus L (**2204**)	dsRNA	785/82.6	1298/70.2	105/31	16/11.8
	Red clover powdery mildew-associated totivirus 7 (**41**)	dsRNA		4/12		37/3.8
	Diatom colony-associated dsRNA virus 3 (**27**)	dsRNA			27/5.2	
	Diatom colony-associated dsRNA virus 16 (**17**)	dsRNA			17/7.3	
***Paramoxyviridae***	HPIV-2 (**384**)	ssRNA	384/34.5			
	HPIV-3 (**305**)	ssRNA		295/35.7		10/4.7
***Coronaviridae***	Human coronavirus NL63 (**223**)	ssRNA	223/10.7			
***Retroviridae***	Feline leukaemia virus (**4**)	ssRNA	4/6.1			
	Human endogenous retrovirus K113 (**126**)	ssRNA-RT	60/8.1	7/1.5	59/17.8	
	Equine infectious anaemia virus (**86**)	ssRNA	40/7.9	46/5.4		
	Reticuloendotheliosis virus (**136**)	ssRNA		75/8.5	61/9.6	
	RD114 Retrovirus (**13**)	ssRNA			13/5.7	
***Partitiviridae***	Ustilaginoidea virens partitivirus 2 (segment RNA 1) (**249**)	dsRNA	249/16.5			
	Fusarium poae virus 1 (segment 1) (**14**)	dsRNA		14/7		
***Chrysoviridae***	Penicillium chrysogenum virus segment 2 (**6**)	dsRNA	6/9.25			
***Picobirnaviridae***	Otarine picobirnavirus (segment 2) (**1551**)	dsRNA			1514/69	37/30.5
***Endornaviridae***	Phaseolus vulgaris alphaendornavirus 1 (**174**)	ssRNA			174/32.9	
	Phaseolus vulgaris alphaendornavirus 2 (**93**)	ssRNA			93/18.7	
***Herpesviridae***	Saimiriine gamma herpesvirus 2 (**2**)	dsDNA			2/0.25	

HRV-C: human rhinovirus C; HRV-A: human rhinovirus A; HRV-B: human rhinovirus B; HPIV-2: human parainfluenza virus 2; HPIV-3: human parainfluenza virus 3.

**Table 5:** Viral family, genus, reads numbers, and genome structure of viruses detected in the respiratory sample pools of the non-SARI group during winter period. **Number of reads/coverage %**

Family	Viral Genus/Read Number	Genome Structure	E2	F2	G2
*Retroviridae*	Equine infectious anaemia virus **(209)**	ssRNA	36/11.2		173/16.3
*Paramoxyviridae*	HPIV-3 **(9)**	ssRNA	9/1.0		
*Bromoviridae*	Ageratum latent virus (**82**)	ssRNA		82/18.3	
	Parietaria mottle virus (segment RNA 2) (**63**)	ssRNA		63/11.9	
*Reoviridae*	Rotavirus A (segment 1) (**17**)	dsRNA			17/13.3
	Rotavirus A (segment 2) (**45**)	dsRNA			45/40.9
	Rotavirus A (segment 6) (**5**)	dsRNA			5/16.7
	Bat Rotavirus (segment 4) (**15**)	dsRNA			15/19
	Rotavirus A (segment 3) (**6**)	dsRNA			6/11
*Picornaviridae*	HRV-C **(1)**	ssRNA		1//1.9	
	HRV-A **(17)**	ssRNA	17/2.8		

HRV-C: human rhinovirus C; HRV-A: human rhinovirus A; HPIV-3: human parainfluenza virus 3.

## Data Availability

The data presented in this study are available in the supplementary information.

## Supplementary Materials

The following supporting information can be downloaded at: https://www.mdpi.com/article/10.3390/v14112516/s1, Table S1: Pooling criteria for the SARI and non-SARI samples in winter and summer; Table S2: Characteristics of the Genome Detective analysis tool; Table S3: Demographic and clinical data of children recruited for the study; Table S4: Detected bacteriophages across all sample pools in both seasons; Table S5: Viral recovery and coverage of the included positive controls; Figure S1: Viral genome coverage and sequencing depth as retrieved from Genome Detective web result. The two representative viruses are (a) SARS-Co-V-2 (b) rhinovirus A; Figure S2: Complete accession numbers of the 100 rhinovirus A sequences retrieved from GenBank.
