# Supplementary material for: Metagenomic Analysis of Respiratory RNA Virome of Children with and without Severe Acute Respiratory Infection from the Free State, South Africa during COVID-19 Pandemic Reveals Higher Diversity and Abundance in Summer Compared with Winter Period
Source: Viruses. 2022 Nov 14;14(11):2516. doi: 10.3390/v14112516 (PMC9692838; doi:10.3390/v14112516)
Supplement: Supplementary file 1 [file viruses-14-02516-s001.zip › viruses-2019987-supplementary.pdf]

Table S1. Pooling criteria for the SARI and Non-SARI samples in winter and summer.

**Summer samples with SARI**	
**Pool Number**	**Timelines during which samples were collected**
n=3 (A1)	Before the detection of first COVID-19 case in the country (Between January to February 2020)
n=8 (B1)	Between COVID lock-down declaration through first wave (End of March 2020 -November 2020)
n=23 (C1)	During second wave of COVID-19. (December 2020–February 2021)
n=55 (D1)	Between second and third waves (February 2021–April 2021)
n=16 (E1)	Early third wave of COVID-19 (May 2021)
**Summer samples without SARI**	
**Pool Number**	**Timeline during which samples were collected**
n=6 (A2)	Between COVID-19 lockdown declaration through first wave (End of March 2020 -November 2020)
n=16 (B2)	During second wave of COVID-19 (December 2020–February 2021)
n=38 (C2)	Between second and third waves of COVID-19 (February 2021–April 2021)
n=20 (D2)	Early third wave of COVID-19 May 2021
**Winter sample with SARI**	
**Pool Number**	**Timeline during which samples were collected**
n=15 (E1)	May/June
n=14 (F1)	July
n=15 (G1)	July/August
**Winter sample without SARI**	
**Pool Number**	**Timelines during which samples were collected**
n=20 (E2)	May/June
n=20 (F2)	July
n=19 (G2)	July/August

Table S2. Characteristics of Genome detective analysis tool.

Classification tool	Genome detective v 2.40
Usage	Patient care
In-house/ commercially available	Commercial
Local/web-based	Web based
De novo assembly	Yes
Alignment of NT/ AA	NT/AA
Database used by participant viral/bacterial (version)	Viruses; based on RefSeq (filtering: Swissprot Uniref 90) v2018-
Paired reads as input option	Yes
Trimming and QC tools	Trimmomatic and FastQC
Exclusion of human reads	Yes
Output type	Web interface, interactive, and Excel
Visualization of genome coverage	Yes
Computational time for analysis per sample (CPU/RAM)	-10 nm (web-based)
Cut-off for defining positive result used	≥3 regions, distributed
Confirmatory analysis required for clinical reporting	Undisclosed
Adapted study specific cut off for defining detection.	≥1 read

Table S3. Demographic and clinical data of children in the SARI and Non-SARI group recruited for the study.

	SARI (n=149)	Non-SARI (n=139)
**Season**		
Summer	105	80
Winter	44	59
**Sex n (%)**		
Male	93 (62.4%)	82 (59.0%)
Female	56 (37.6%)	57 (41.0%)
**Age**		
Median in months	14.5	19
**SARI clinical details n (%)**		
Cough	149 (100%)	N/A
Measured fever ≥38°C	149 (100%)	N/A
Need for oxygen	66 (44.2%)	N/A
Feeding difficulty	23 (15.4%)	N/A
Chest indrawing	54 (36.2%)	N/A
Hospitalization	149 (100%)	N/A
**SARI suspected diagnoses n (%)**		
**Bronchiolitis**	33	N/A
**Pneumonia**	52	N/A
**bronchopneumonia**	24	N/A
**Unspecified**	30	N/A
**Epiglottitis**	1	N/A
**Asthma and pneumonia**	5	N/A
**Viral meningitis**	1	N/A
**Jeune dystrophy syndrome**	1	N/A
**Croup grade 3**	2	N/A
**Other SARI parameters n (%)**		
Asthmatic	5 (3.3%)	N/A
HIV infected	27 (18.1%)	N/A
ICU admission	0 (0%)	N/A
Household member smokes	12 (8.0%)	N/A
Attends creche	19 (12.7%)	N/A
**Non-SARI clinical details n (%)**		
Convulsion and seizure	N/A	9 (6.4%)
Gastroenteritis	N/A	19 (13.6%)
Acute malnutrition	N/A	22 (15.7%)
Fever	N/A	5 (3.5%)

Epilepsy	N/A	3 (2.14%)
Heart problem	N/A	2 (1.4%)
Meningitis	N/A	3 (2.14%)
Dog bite	N/A	2 (1.4%)
Paraffin ingestion	N/A	4 (2.8%)
Neurological problem	N/A	1 (0.71%)
Nephrotic syndrome	N/A	1 (0.71%)
Abscess	N/A	3 (2.14%)
Rash	N/A	1 (0.71%)
Gastroenteritis and malnutrition	N/A	2 (1.4%)
Eczema	N/A	4 (2.8%)
Hoarseness of the voice	N/A	1 (0.71%)
Injury	N/A	14 (10%)
Skin infection	N/A	3 (2.14%)
Diarrhoea and vomiting	N/A	2 (1.4%)
Fractured bone	N/A	2 (1.4%)
Anaemia	N/A	2 (1.4%)
Burns	N/A	7 (5%)
Ear infection	N/A	2 (1.4%)
Vomiting	N/A	2 (1.4%)
Dehydration	N/A	2 (1.4%)
Neonatal encephalopathy	N/A	1 (0.71%)
Dental problem	N/A	5 (3,5%)
Coin ingestion	N/A	2 (1.43%)
Smoke inhalation	N/A	1 (0.71%)
Diarrhoea	N/A	2 (1.4%)
Epistaxis	N/A	3 (2.14%)
Swollen knee	N/A	1 (0.71%)
Electrical shock	N/A	1 (0.71%)
Eye pain	N/A	2 (1.43%)
Jaundice	N/A	1 (0.71%)
Cellulitis	N/A	1 (0.71%)
Swollen neck	N/A	1 (0.71%)
**Other Non-SARI parameters n (%)**		
Household member smokes	N/A	14(10%)
Attends creche	N/A	11(7.8%)
HIV infected	N/A	12 (8.5%)

Table S4. Detected bacteriophages across all sample pools in both seasons.

Detected bacteriophages in both SARI and non-SARI pools across both seasons	Total number of reads
Escherichia phage	644
Enterobacteria phage	961
Streptococcus phage	832
Klebsiella phage	468
Staphylococcus phage	1113
Stx2-converting phage	838
Lactococcus phage	896
Vibrio phage	139
Salmonella phage	655
Proteus phage	937
Aggregatibacter phage	166

Table S5. Viral recovery and coverage of the included positive controls.

Family	Genus	Coverage	Depth	Reads	NT identity	AA Identity
Coronaviridae	SARS-CoV-2	68.9%	78.1%	9256	99.9%	99.5%
Picornaviridae	Rhinovirus A	93.6%	28.5%	1091	86.6%	89.8%

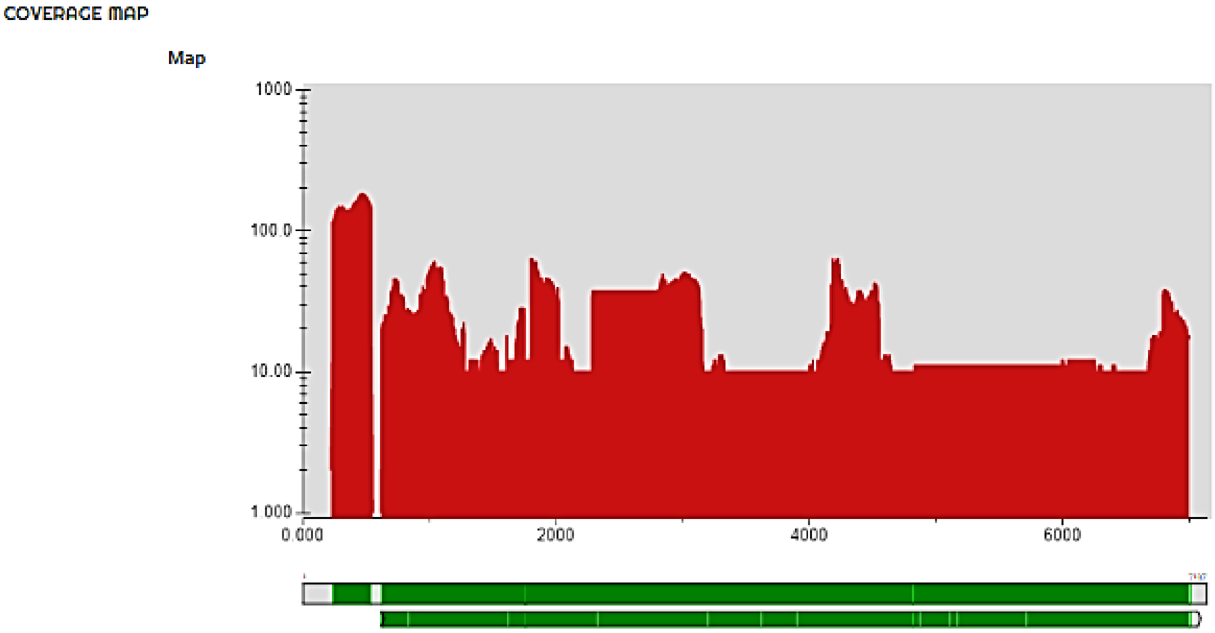

(a)

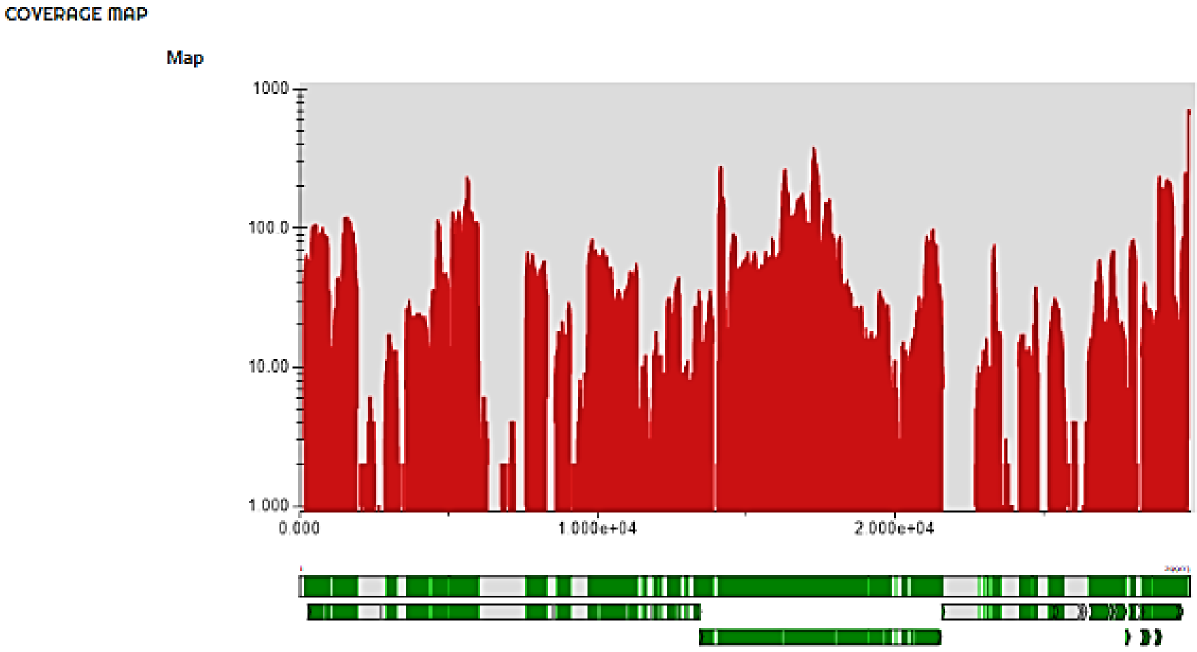

(b)

Figure S1. Viral genome coverage and sequencing depth as retrieved from genome detective web result. The two representative viruses are **(a)** SARS-Co-V-2 **(b)** Rhinovirus A.

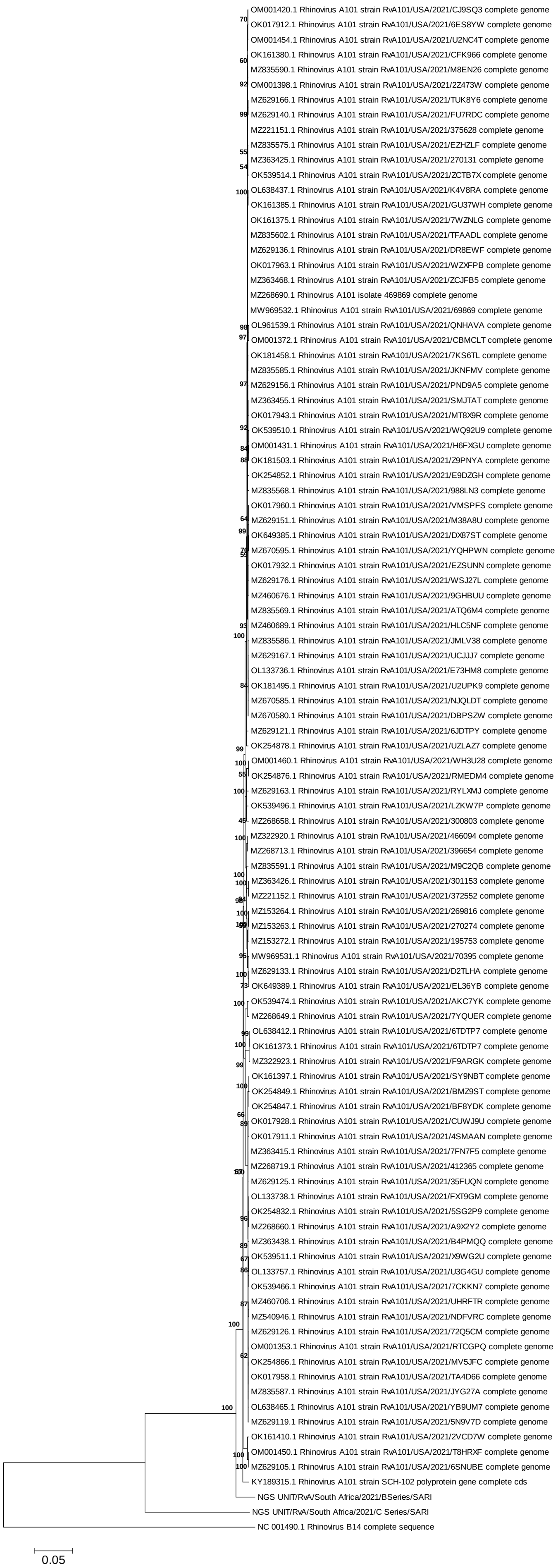

Figure S2. Complete 100 rhinovirus A sequences retrieved from GenBank.
